# An Update of a Clinical Practice Guideline for the Management of Patients With Acute Spinal Cord Injury: Recommendations on the Role and Timing of Decompressive Surgery

**DOI:** 10.1177/21925682231181883

**Published:** 2024-03-25

**Authors:** Michael G. Fehlings, Lindsay A. Tetreault, Laureen Hachem, Nathan Evaniew, Mario Ganau, Stephen L. McKenna, Chris J. Neal, Narihito Nagoshi, Vafa Rahimi-Movaghar, Bizhan Aarabi, Christoph P. Hofstetter, Valerie ter Wengel, Hiroaki Nakashima, Allan R. Martin, Steven Kirshblum, Ricardo Rodrigues Pinto, Rex A. W. Marco, Jefferson R. Wilson, David E. Kahn, Virginia F. J. Newcombe, Carl M. Zipser, Sam Douglas, Shekar N. Kurpad, Yi Lu, Rajiv Saigal, Uzma Samadani, Paul M. Arnold, Gregory W. J. Hawryluk, Andrea C. Skelly, Brian K. Kwon

**Affiliations:** 1 Department of Surgery, Division of Neurosurgery and Spine Program, 7938 University of Toronto, Toronto, ON, Canada; 2 Division of Neurosurgery, Krembil Neuroscience Centre, Toronto Western Hospital, 7989 University Health Network, Toronto, ON, Canada; 3 Institute of Medical Science, 7938 University of Toronto, Toronto, ON, Canada; 4 Department of Neurology, NYU Langone Medical Center, New York, NY, USA; 5 Department of Surgery, Orthopaedic Surgery, McCaig Institute for Bone and Joint Health, Cumming School of Medicine, 2129 University of Calgary, Calgary, AB, Canada; 6 Nuffield Department of Clinical Neurosciences, University of Oxford, Oxford, UK; 7 Department of Neurosurgery, 6397 Oxford University Hospitals NHS Foundation Trust, Oxford, UK; 8 Department of Neurosurgery, 6429 Stanford University, Stanford, CA, USA; 9 Department of Surgery, Uniformed Services University, Bethesda, MD, USA; 10 Department of Orthopaedic Surgery, 38084 Keio University School of Medicine, Tokyo, Japan; 11 Sina Trauma and Surgery Research Center, 48439 Tehran University of Medical Sciences, Tehran, Iran; 12 Department of Neurosurgery, 1479 University of Maryland School of Medicine, Baltimore, MD, USA; 13 Department of Neurological Surgery, 7284 University of Washington, Seattle, WA, USA; 14 Department of Neurosurgery, Amsterdam UMC VUMC Site, Amsterdam, Netherlands; 15 Department of Orthopedic Surgery, 36590 Nagoya University Graduate School of Medicine, Nagoya, Japan; 16 Department of Neurological Surgery, University of California-Davis, Sacramento, CA, USA; 17 Kessler Institute for Rehabilitation, 12286 Rutgers New Jersey Medical School, Newark, NJ, USA; 18 Spinal Unit (UVM), 112085 Centro Hospitalar Universitário de Santo António, Hospital CUF Trindade, Porto, Portugal; 19 Department of Orthopedic Surgery, 570987 Houston Methodist Hospital, Houston, TX, USA; 20 Department of Medicine, University Division of Anaesthesia and PACE, 2152 University of Cambridge, Cambridge, UK; 21 Spinal Cord Injury Center, 31031 Balgrist University Hospital, Zurich, Switzerland; 22 Praxis Spinal Cord Institute, Vancouver, BC, Canada; 23 Department of Neurosurgery, 5506 Medical College of Wisconsin, Milwaukee, WI, USA; 24 Department of Neurosurgery, Harvard Medical School, 1861 Brigham and Women’s Hospital, Boston, MA, USA; 25 Department of Surgery, Minneapolis Veterans Affairs, Minneapolis, MN, USA; 26 Department of Neurosurgery, University of Illinois Champaign-Urbana, Urbana, IL, USA; 27 Neurosurgery, 1077 Cleveland Clinic Akron General Hospital, Akron, OH, USA; 28 Aggregate Analytics, Inc., Fircrest, WA, USA; 29 Department of Orthopaedics, 8166 University of British Columbia, Vancouver, BC, Canada; 30 International Collaboration on Repair Discoveries (ICORD), 8166 University of British Columbia, Vancouver, BC, Canada

**Keywords:** spinal cord injury, decompression, trauma, clinical practice guideline, timing of surgery, neurological outcomes, early surgery

## Abstract

**Study Design:**

Clinical practice guideline development.

**Objectives:**

Acute spinal cord injury (SCI) can result in devastating motor, sensory, and autonomic impairment; loss of independence; and reduced quality of life. Preclinical evidence suggests that early decompression of the spinal cord may help to limit secondary injury, reduce damage to the neural tissue, and improve functional outcomes. Emerging evidence indicates that “early” surgical decompression completed within 24 hours of injury also improves neurological recovery in patients with acute SCI. The objective of this clinical practice guideline (CPG) is to update the 2017 recommendations on the timing of surgical decompression and to evaluate the evidence with respect to ultra-early surgery (in particular, but not limited to, <12 hours after acute SCI).

**Methods:**

A multidisciplinary, international, guideline development group (GDG) was formed that consisted of spine surgeons, neurologists, critical care specialists, emergency medicine doctors, physical medicine and rehabilitation professionals, as well as individuals living with SCI. A systematic review was conducted based on accepted methodological standards to evaluate the impact of early (within 24 hours of acute SCI) or ultra-early (in particular, but not limited to, within 12 hours of acute SCI) surgery on neurological recovery, functional outcomes, administrative outcomes, safety, and cost-effectiveness. The GRADE approach was used to rate the overall strength of evidence across studies for each primary outcome. Using the “evidence-to-recommendation” framework, recommendations were then developed that considered the balance of benefits and harms, financial impact, patient values, acceptability, and feasibility. The guideline was internally appraised using the Appraisal of Guidelines for Research and Evaluation (AGREE) II tool.

**Results:**

The GDG recommended that early surgery (≤24 hours after injury) be offered as the preferred option for adult patients with acute SCI regardless of level. This recommendation was based on moderate evidence suggesting that patients were 2 times more likely to recover by ≥ 2 ASIA Impairment Score (AIS) grades at 6 months (RR: 2.76, 95% CI 1.60 to 4.98) and 12 months (RR: 1.95, 95% CI 1.26 to 3.18) if they were decompressed within 24 hours compared to after 24 hours. Furthermore, patients undergoing early surgery improved by an additional 4.50 (95% 1.70 to 7.29) points on the ASIA Motor Score compared to patients undergoing surgery after 24 hours post-injury. The GDG also agreed that a recommendation for ultra-early surgery could not be made on the basis of the current evidence because of the small sample sizes, variable definitions of what constituted ultra-early in the literature, and the inconsistency of the evidence.

**Conclusions:**

It is recommended that patients with an acute SCI, regardless of level, undergo surgery within 24 hours after injury when medically feasible. Future research is required to determine the differential effectiveness of early surgery in different subpopulations and the impact of ultra-early surgery on neurological recovery. Moreover, further work is required to define what constitutes effective spinal cord decompression and to individualize care. It is also recognized that a concerted international effort will be required to translate these recommendations into policy.

## Summary of Recommendations

**Population Description:** Adult patients with acute spinal cord injury (SCI).

**Key Question 1:** Should we recommend early decompressive surgery (≤24 hours after injury) for adult patients with acute SCI regardless of injury severity and neurological level?

**Recommendation***:* We recommend that early surgery be offered as an option for adult patients with acute SCI regardless of level.

**Quality of Evidence:** Moderate.

**Strength of Recommendation:** Strong.

**Population:** Adult patients with acute SCI.

**Key Question 2:** Should we recommend ultra-early decompressive surgery for adult patients with acute SCI regardless of injury severity and neurological level?

**Statement:** A recommendation for ultra-early surgery could not be made on the basis of the current evidence because of the small sample sizes, variable definitions of what constituted ultra-early, and the inconsistency of the evidence.

## Introduction

Traumatic SCI can result in devastating motor, sensory, and autonomic impairment, as well as loss of independence and reduced quality of life.^1^ The acute and long-term management of SCI requires substantial health care resources and can impose significant financial burden on patients, families, and communities.^2^ The treatment of SCI has evolved over time due to the emergence of several preclinical and clinical studies on injury mechanisms, disease pathophysiology, the role of surgical intervention, and novel neuroprotective strategies.

Primary injury refers to the initial mechanical forces applied to the spinal cord by vertebral fractures, disc debris, or disruption of the supporting ligaments.^3,4^ Within minutes to hours of injury, significant pathophysiological changes occur that can damage the ascending and descending neural pathways and induce spinal shock.^5,6^ These changes include vasospasm, ischemia, disruption of the blood–spinal cord barrier, ion imbalance, and accumulation of neurotransmitters.^5^ Secondary injury begins within minutes of the initial trauma and may cause progressive spinal cord damage for weeks to months through demyelination, Wallerian degeneration, and formation of a glial scar.^4^ Preclinical evidence suggests that early decompression of the spinal cord may help to limit this secondary injury, reduce damage to the neural tissue, and improve functional outcomes.^7–9^ This concept of timely surgical intervention has also been investigated by a number of clinical trials and cohort studies, allowing for the development of an evidence-based clinical practice guideline (CPG) in 2017.^10^

This 2017 CPG provided recommendations for the timing of surgical intervention in patients with SCI, using 24 hours as the threshold between early and late decompression.^10^ Based on a systematic review of the literature and a guideline development framework, a weak recommendation was formulated to suggest early surgery be offered as an option for adult SCI patients regardless of injury level.^10,11^ Since the publication of this CPG, several studies have emerged that assess the impact of early vs late surgical decompression on neurological recovery, functional outcomes, and quality of life.^12–21^ Given the availability of new evidence, it is advised that the CPG from 2017 be updated as the results of recent studies may change the strength of previous recommendations and impact clinical decision making. Furthermore, as care pathways for patients with SCI become more streamlined, it may be possible to intervene even earlier, especially if beneficial. It is therefore the endeavor of this CPG to explore different time cutoffs (eg, <4, <8, or <12 hours) in order to establish the ideal timing of surgical intervention in terms of impact on outcome, cost-effectiveness, and feasibility.

The overarching aim of this CPG is to standardize care, improve outcomes, and reduce morbidity and mortality in patients with SCI by creating recommendations on the timing of surgical intervention. The specific aims of this CPG are to address the following 2 questions:

1. Should we recommend early decompressive surgery (≤24 hours after injury) for adult patients with acute SCI regardless of injury severity and neurological level?
2. Should we recommend ultra-early decompressive surgery for adult patients with acute SCI regardless of injury severity and neurological level?

## Methods

The development of this CPG was sponsored by AO Spine and Praxis Spinal Cord Institute. A multidisciplinary guideline development group (GDG) was established that consisted of neurosurgeons, orthopedic surgeons, neurologists, critical care specialists, emergency medicine doctors, first responders, physical medicine and rehabilitation professionals, as well as individuals living with SCI. Each member of the GDG was required to disclose both financial and intellectual conflicts of interest. Methodologists at Aggregate Analytics Inc were responsible for providing the expertise for both the systematic review of the literature and the development of the CPG. The protocol for this CPG was formulated using the Conference on Guideline Standardization (COGS) framework and the Checklist for Reporting the Updating Process (CheckUp) and is published in a separate article in this focus issue.^22,23^

A systematic review was conducted based on accepted methodological standards to address the following key questions:

1. What is the evidence for the effectiveness of early decompression (≤24 hours) compared with late decompression (>24 hours) or conservative therapy based on clinically important change in neurological status? What is the effectiveness of ultra-early decompression compared with other “early” time frames up to 24 hours (eg, but not limited to <12 hours vs ≥ 12 hours but <24 hours)?
2. Does timing of decompression influence other functional outcomes or administrative outcomes?
3. What is the safety profile of early decompression compared with late decompression?
4. Does early decompression have differential efficacy or safety in specific subgroups of patients?
5. What is the cost-effectiveness of early decompression compared with late decompression?

The approach outlined by the Grading of Recommendation, Assessment, Development, and Evaluation (GRADE) Working Group was used to rate the overall strength of evidence across studies for each primary outcome.^24^ The results of the systematic review and the evidence tables were presented to the GDG. The GRADE “evidence-to-recommendation” framework was used to support the guideline development process and ensure that each recommendation considered other factors, including benefits and harms, resource use, impact on health inequities, acceptability, and feasibility.^25–27^ The last step in the framework was to balance the desirable and undesirable consequences and determine the strength of each recommendation. The Appraisal of Guidelines for Research and Evaluation (AGREE) II was used to internally appraise the CPG.^28^ Finally, this document was distributed to a multidisciplinary group of clinicians as well as prominent societies in spine surgery for external review. A complete summary of the methods used to create this CPG is provided in a separate article in this focus issue entitled “An Overview of the Methodology Used to Develop Clinical Practice Guidelines for the Management of Acute and Intraoperative Spinal Cord Injury.”

## Clinical Recommendations

### Part 1. Timing of Decompressive Surgery (≤24 Hours After Injury) in Adult Patients With Acute Spinal Cord Injury

**Population Description:** Adult patients with acute SCI.

**Key Question 1:** Should we recommend early decompressive surgery (≤24 hours after injury) for adult patients with acute SCI regardless of injury severity and neurological level?

**Recommendation:** We recommend that early surgery be offered as an option for adult patients with acute SCI regardless of level.

**Quality of Evidence:** Moderate.

**Strength of Recommendation***:* Strong.

#### Evidence Summary

A systematic review of the literature was conducted in order to summarize and appraise the available evidence on the effectiveness and safety of early (≤24 hours post-injury) surgical decompression compared to late (>24 hours post-injury) surgical decompression after acute SCI.

*Evidence Related to Benefits*. Nine studies reported on change in ASIA Impairment Scale (AIS) at 6 or 12 months following injury.^13,15,16,19–21,29–31^ Of these, 5 studies compared the rate of improvement by ≥ 2 AIS grades at 6 months between patients undergoing early vs late surgery.^20,21,29,30,32^ Based on pooled estimates, patients were 2.76 (95% CI 1.60 to 4.98) times more likely to recover by ≥ 2 AIS grades if they were decompressed within 24 hours compared to after 24 hours. Four studies evaluated the impact of timing of surgery on improving by ≥ 2 AIS grades at 12 months.^13,15,16,19^ Similarly, patients were approximately 2 (95% CI 1.26 to 3.18) times more likely to exhibit a ≥2 grade improvement on the AIS at 12 months if treated within 24 hours compared to after 24 hours. The overall strength of evidence for these outcomes (≥2 grade improvement in AIS at 6 and 12 months) was moderate.

Five studies explored the change in ASIA motor score (AMS) between individuals operated on early vs late.^12,14,16,31,32^ The results from the 2 studies that reported on AMS at 6 months could not be pooled due to substantial clinical heterogeneity and limited reporting of data.^31,32^ A single study identified that patients undergoing early (<24 hours post-injury) surgery for acute central cord syndrome (CCS) gained an additional 7.47 points on the AMS compared to those treated late.^31^ A second study demonstrated that early surgery confers a 13-point improvement in AMS in individuals with cervical, thoracic, or lumbosacral SCI.^32^ Based on the results of these 2 studies, there is very low evidence that early surgery improves AMS in the short term. The remaining 3 studies explored change in AMS at >6–12 months and were evaluated in a meta-analysis.^14,16,31^ Based on pooled estimates, patients undergoing early surgery improved by an additional 4.50 (95% 1.70 to 7.29) points on the AMS compared to patients treated late. The overall strength of evidence was moderate for this outcome. In summary, the evidence is very low that early surgery impacts AMS in the short term (<6 months) but moderate that it results in motor improvement in the longer term (>6–12 months).

A single prospective observational study evaluated the impact of early decompression on functional independence measure (FIM) motor and total scores.^31^ Based on the results, patients undergoing early surgery for acute CCS were more likely to exhibit improvements in FIM total scores (MD 7.79, 95% CI .09 to 15.49, *P* = .0474) than those treated late.^31^ However, the overall level of evidence for this outcome was rated as very low due to serious imprecision in the estimate of effect.

*Evidence Related to Harms*. Several studies discussed rates of complications between patients treated surgically within 24 hours of injury and those decompressed after 24 hours.^13,15,16,20,21,29,30,33^ Based on the results of the systematic review, there were no statistically significant differences in critical harm outcomes between early and late surgery groups. Furthermore, rates of the majority of reported complications tended to be higher in the late surgery group, including mortality, decubitus ulcers, cardiopulmonary events, and need for tracheostomy.^15,16,20,29,30,33^ In contrast, the frequency of neurological deterioration and sepsis secondary to systemic infection tended to be higher in the early surgery groups.^13,29,30^ Unfortunately, the majority of studies were underpowered to detect a difference in rates of complications based on timing of surgery.

Based on a single study and moderate evidence, there was no difference in the rate of major complications between early (24.2%) and late surgery (30.5%, RR .79, 95% CI .55 to 1.14).^29^ Two randomized controlled trials and four observational studies evaluated mortality rates in patients treated early vs late.^16,20,29,30,33^ Mortality was uncommon and did not significantly differ between groups: 2.6% in the early group and 3.6% in the late group (RR .68, 95% CI .29 to 1.50). Although results across studies were consistent, the overall quality of evidence was rated as low due to moderate risk of bias and imprecision. Three studies examined differences in rates of fixation or construct failure and one study assessed differences in rates of screw revision or pull-out based on timing of surgery.^16,20,29^ Based on low evidence, there were no differences in rates of surgical-device related complications between patients decompressed early compared to those treated late (fixation or construct failure: 1.5% vs 1.4%, RR 1.21, 95% CI .21 to 5.87; revision of lateral screws or screw pull-out: 8.1% vs 8.3%, RR .97, 95% CI .21 to 4.51). Based on low evidence, there were no differences in the frequency of sepsis between patients treated early (1.7%) compared to late (.8%, RR 1.96, 95% CI .50 to 7.60).^15,29^ Four studies assessed the rate of decubitus or pressure ulcers based on timing of surgery and concluded that patients treated early had fewer decubitus or pressure ulcers than those treated late (3.8% vs 6.9%, RR .81, 95% CI 0.46 to 1.37).^15,16,20,33^ However, this difference did not reach statistical significance and the overall rating of the evidence was low due to moderate risk of bias. Three studies reported no difference in the rate of neurological deterioration following early (4.7%) vs late (.7%) surgery in patients with cervical SCI (RR 3.51, 95% CI .73 to 17.23).^13,29,30^ The overall rating of the evidence for this outcome was very low. Finally, based on the results of single studies and low evidence, there were fewer cardiopulmonary complications (17.6% vs 25.9%, RR .68, 95% CI .44 to 1.04) and tracheostomies (45% vs 55%, RR .82, 95% CI .54 to 1.25) in the early surgery group compared to the late surgery group; however, this difference did not reach statistical significance.^20,29^

#### Rationale for Recommendation

The outcomes ranked as critical for decision making were improvement of AIS by ≥ 2 grades, AMS, and FIM as well as any major complications, mortality, surgical device-related complications, sepsis secondary to systemic infection, pressure ulcers, neurological deterioration, cardiopulmonary events, and need for tracheostomy. It was also decided that the proposed recommendation would not distinguish between patients with complete and incomplete injuries or specify the level of injury. The reason behind this decision was based on the results of the meta-analysis that assessed whether injury severity or level modified the effects of timing of surgery with respect to improvement by ≥ 2 grades on the AIS. Based on the results, the risk ratio estimates in both the complete and incomplete injury groups suggested benefit of early decompression. Furthermore, the confidence intervals for the estimates of effect overlapped, and the test for interaction was not significant. Based on these results, it can be concluded that injury severity does not modify the treatment effect of early surgery in patients with SCI. Unfortunately, data was insufficient to hypothesize whether the level of SCI alters the effect of early surgery. However, given the similarity of point estimates and overlapping confidence intervals when comparing the impact of early surgery on outcomes across injury levels, there is likely no modification of treatment effect. Furthermore, the risk ratios suggest potential benefit of early surgery across all levels of SCI.

An important first step in the guideline development process was to identify the minimum clinically important difference (MCID) for each critical outcome. The MCID refers to the smallest change in a score that a patient would perceive as beneficial or meaningful.^34,35^ It is postulated that neurological improvement is only meaningful to a patient living with SCI if it translates to functional benefits and enhancement in quality of life. Furthermore, the MCIDs for neurological recovery and functional outcomes are likely dependent on the level and severity of SCI. The International Standards for Neurological Classification of SCI Patients (ISNCSCI) is the preferred tool to assess the severity and level of SCI and quantify motor and sensory dysfunction.^36–38^ It is an ordinal scale that is not linear, meaning that a one-grade change carries different value depending on patient characteristics.^39^ Using distribution-based methods, the MCID for the upper extremity motor score (UEMS), lower extremity motor score (LEMS), and total motor score (TMS) was computed as 2.72, 3.66, and 4.48, respectively, in a population of acute and subacute traumatic or nontraumatic SCI.^40^ For individuals with cervical SCI, the MCID for the UEMS ranged from 1.9 for AIS D patients to 2.91 for ASIA A patients.^40^ Similarly, the MCID of the LEMS was lower in patients with AIS D injuries (1.5) than individuals with more severe AIS grades (2.45 AIS C and 2.35 AIS B). Finally, the MCID of the TMS was 2.22 in AIS D, 4.16 in AIS C, 3.23 in AIS B, and 2.91 in AIS A patients.^40^ In other studies, the minimum detectable change of the UEMS was estimated to be 2 or 3,^41,42^ with 6 points being the average spontaneous improvement seen in acute cervical sensorimotor complete injuries.^39^ Despite extensive efforts to define the MCID for UEMS, LEMS, and TMS in SCI patients, it remains a clinical challenge because the myotomal distribution of improvement may be more important than the absolute change in the motor score. For example, trace flicker (score of 1) in 2 myotomes on each side of the body is not as clinically meaningful to a patient as obtaining a 4-point improvement across 2 myotomes in a single extremity. In a study by Scivoletto et al (2013), the effect-size based estimate for a substantial change was also calculated.^40^ Based on their results, a 7.45-point change was considered substantial for UEMS, 8.4 for LEMS, and 10.65 for TMS. These results were presented to the GDG who were asked to vote on the MCID for AMS using either clinical expertise or personal experiences. Forty-five percent of participants voted that any change in AMS was meaningful for individuals living with SCI, while 18% stated 2 points, 5% selected 3 points, 23% agreed 4 points, and 9% chose 5 points as the likely MCID. While there was a lack of consensus, it is important to note that the majority of the GDG (91%) agreed that an improvement in AMS of 4 points (and sometimes less than this) is likely to be clinically meaningful for patients with SCI. While this method for defining what constituted a “clinically meaningful change” may not be grounded in a formal statistical analysis of empirical evidence, it did incorporate the opinions of a diverse set of stakeholders, including individuals living with SCI.

A 2-grade improvement in the AIS was considered a critical outcome for decision making, while a 1-grade improvement was deemed important but not critical. The GDG agreed that a 2-grade improvement is likely to be clinically meaningful in the majority of patients. While a 1-grade improvement may still lead to some functional benefits in select patients,^43^ it does not reliably translate to improved functional status, reduced disability, or enhanced quality of life. For example, a change in AIS from A to B will likely not significantly impact overall function, nor would improving from ASIA B to C if the only gain is a trace flicker of movement in the lower extremities. In patients with ASIA C or D injuries, however, a 1-grade change in AIS may translate to significant improvements in function. Given the variable clinical significance of a 1-grade change in AIS, the GDG agreed that a 2-grade improvement should be the main outcome driving the clinical recommendation.

As highlighted above, the overall quality of evidence for these critical outcomes was rated as “moderate,” “low,” or “very low.” The GDG agreed that the main outcomes driving the recommendation on timing of surgery are improvement in AIS by ≥ 2 grades and improvement in AMS. In contrast, the GDG acknowledged that outcomes related to harm should not drive the recommendation as early surgery does not increase the risk of major complications or mortality, and because the majority of studies comparing early and late surgery were underpowered to detect any difference in rates of adverse events. The GDG also agreed that neurological outcomes at >6 to 12 months are more relevant than at <6-month follow-up as patients often continue to demonstrate improvement up to and sometimes after a year following injury. Based on these discussions, 95% of the GDG voted that the overall certainty of evidence was moderate.

The GDG agreed that there was either probably no (65%) or no (30%) important uncertainty or variability with respect to how much key stakeholders value the main outcomes. Based on professional opinion, it is likely that clinicians and patients would similarly value the outcomes related to neurological and functional improvement, as well as serious adverse events. While these outcomes would also be valued by payers, this stakeholder group would also value length of stay, resource use, and cost-effectiveness.

The anticipated desirable effects of early surgical decompression include clinically meaningful improvements in AIS and AMS. Based on moderate evidence, patients were approximately 2 times more likely to improve by ≥ 2 AIS grades at 6 months (RR 2.76, 95% CI 1.60 to 4.98) or 12 months (1.95, 95% CI 1.26 to 3.18) if they were decompressed within 24 hours of injury compared to after 24 hours. An effect size greater than 2 is considered large. In addition, moderate evidence suggested that early surgery resulted in a 4.50 (95% CI 1.70 to 7.29) point improvement in AMS at > 6 to 12 months.

Ninety percent of the GDG agreed that the anticipated desirable effects of early surgical decompression were either moderate (48%) or large (43%). Participants who voted “moderate” commented that while a 2-point improvement in AIS and a 4.5 improvement in AMS are likely clinically meaningful, the MCIDs of these outcome tools have not been rigorously assessed in a traumatic SCI population. Furthermore, the GDG acknowledged that neurological recovery on the AIS and AMS may inconsistently translate to functional benefit and often depends on the myotomal distribution of improvements (as described above) or whether changes are appreciated in the upper or lower extremities. In contrast, participants who selected “large” emphasized that any improvement in neurological function has the potential to significantly impact the quality of life of an individual living with SCI.

The anticipated undesirable effects of early surgical decompression include any major complication, mortality, surgical device-related complications, sepsis secondary to systemic infection, pressure ulcer, neurological deterioration, cardiopulmonary dysfunction, and need for tracheostomy. Based on very low to moderate evidence, there was no difference in the rate of complications between patients treated surgically within 24 hours and those treated after 24 hours. However, most studies were underpowered to detect a difference in rates of complications based on timing of surgery. Furthermore, it is often difficult to distinguish complications associated with the underlying SCI from those related to surgery. Fifty-five percent of the GDG voted that the undesirable effects of early surgery were likely trivial, and 45% agreed they were small. The anticipated undesirable effects were considered trivial not because these complications are trivial but because there are no additional risks following early compared to late surgical intervention. The GDG unanimously agreed that the balance between the desirable and undesirable effects favors early surgical decompression given the potential neurological benefit without an increased risk of complications.

The financial burden of SCI is significant due to costs associated with acute and chronic medical care, in addition to costs related to loss of productivity. Individuals with SCI have a higher rate of rehospitalizations, visits to primary care physicians, and home services than a matched cohort of individuals without SCI.^44,45^ Based on a systematic review of the literature, the mean cost of SCI management in the first year was $119,870 in Canada and between $300,880 and $634,400 in the United States.^46^ Furthermore, based on a study by Krueger et al (2013), lifetime expenses range from $2,105,811 to $3,026,028 CAD for individuals with incomplete and complete tetraplegia, respectively.^2^

Although the financial burden of SCI is well known, there was limited data on whether costs differ based on timing of surgical intervention. A study by Mac-Thiong et al (2012) compared the cost of acute management between individuals receiving early vs late surgery for SCI.^47^ Based on their results, the hospitalization costs were $20,525 ± 13,791 in the <24 hour surgery group and $25,036 ± 17,886 in the ≥24 hour surgery group. There were, however, significant baseline differences confounding these results: patients treated within 24 hours of injury were younger, more likely to have a lower AIS, and more likely to have sustained a thoracolumbar neurological injury.^47^ A cost utility analysis was conducted by Furlan et al (2016) to compare the cost-effectiveness of early (≤24 hours) vs late (>24 hours) surgery in patients with acute traumatic cervical SCI.^48^ In patients with motor complete SCI, the cost of early decompression was $524,483.81 USD/QALY, whereas the cost of late surgery was $544,851.71 USD/QALY. Furthermore, the potential savings of early surgery were estimated to be $58,368,024 USD/QALY. However, using a Monte Carlo simulation, early decompression was more cost-effective 26.3% of the time but less cost-effective 23.4% of the time. These results indicate that no strategy was clearly dominant in motor complete SCI. In a group of patients with motor incomplete SCI, the cost of early decompression was $83,009.19 USD/QALY, whereas the cost of late decompression was $81,233.10 USD/QALY. Early surgery was deemed to be more cost-effective than late surgery with the potential to save $536,217.33 USD/QALY in individuals with motor incomplete SCI. Using Monte Carlo simulation, early decompression was more cost-effective in 32.6% but less cost-effective in 18.2% of patients with motor incomplete SCI. Again, these findings indicate that no strategy is clearly dominant. This study concluded that early decompression is more cost-effective than late decompression in patients with complete and incomplete SCI, even if there was clearly no dominant strategy.^48^ The level of evidence was not graded for these results.

Length of stay was also considered when assessing overall cost and resource requirement. Six studies were identified that compared hospital length of stay between patients treated early vs late for acute SCI.^15,20,21,32,49,50^ In a meta-analysis, pooled estimates across 5 studies indicated that patients undergoing early surgery had a shorter length of stay than those treated late (mean difference −3.52, 95% CI −4.08 to −2.95).^15,20,21,32,50^ The overall level of evidence for this outcome was low. While a shorter length of stay can drastically reduce costs, this difference likely reflects only minor savings when considering the financial burden of SCI. However, institutions often use length of stay as a metric of performance.^51^ Furthermore, a shorter time spent in the hospital reduces the risk of hospital-acquired complications (eg, infection, delirium, falls, pressure ulcers, and respiratory complications), which can further prolong the length of stay, increase cost, and impact outcomes. Fifty-eight percent of the GDG agreed the costs and savings associated with early surgical decompression are negligible given that the resource requirements are similar for early and late surgery. Sixteen percent either selected large (11%) or moderate (5%) savings with early surgical decompression as the neurological improvements associated with early surgery may result in significant lifelong savings. An additional 16% of the group selected moderate costs associated with early surgery, 5% stated the resource requirements vary, and 5% voted that they did not know given the absence of strong literature.

The GDG agreed that the certainty of evidence related to resource requirements was very low (64%) or low (18%). They voted that cost-effectiveness probably favors (64%) or favors (27%) early surgical decompression given that the resource requirement is likely similar between early vs late surgery and because early surgery is associated with improved neurological outcomes and a shorter length of stay.

The votes varied with respect to the impact of a recommendation of early surgery on health inequities: 38% uncertain, 48% probably reduced, 5% reduced, and 10% don’t know. If it is assumed that standardized pathways are implemented in order to ensure direct transfer from the site of injury to a trauma center capable of urgently performing surgical decompression, the GDG agreed that health inequities would probably be reduced. If policy makers funded initiatives to ensure patients with traumatic SCI are triaged appropriately and have better access to early surgical intervention, then there would be less disparity across socioeconomic groups and geographic regions.

The GDG agreed that a recommendation for early surgery would be acceptable to key stakeholders (probably yes: 45%, yes 50%) due to the potential improvement in AIS and AMS with no added risks. The GDG acknowledged that patients with devastating injuries may value small neurological improvements as these may translate to clinically meaningful changes in functional status and quality of life. Furthermore, a recommendation for early surgery would likely be acceptable to payers due to no added costs and the potential for significant long-term savings. The GDG also agreed that a recommendation for early surgery would probably be feasible to implement (probably yes 63%, yes: 26%, and varies: 11%) but that this would depend on the clinical setting, geographic region, and local guidelines.

Considering these factors, 95% of the GDG voted that the desirable consequences clearly outweigh the undesirable consequences in most settings. This consensus led to the formation of a strong recommendation for early decompression in patients with traumatic SCI (100%).

The final wording that a recommendation be made for surgical decompression within 24 hours post-injury was subjected to a final vote of all the GDG members and consensus was achieved for its approval (>90%). There was 1 “dissenting” concern raised in the final voting that the recommendation should be stronger given the quality of the evidence.

### Part 2. Ultra-Early Decompressive Surgery in Adult Patients With Acute Spinal Cord Injury

**Population:** Adult patients with acute SCI.

**Key Question 2:** Should we recommend ultra-early decompressive surgery for adult patients with acute SCI regardless of injury severity and neurological level?

**Statement:** A recommendation for ultra-early surgery could not be made on the basis of the current evidence because of the small sample sizes, variable definitions of what constituted ultra-early, and the inconsistency of the evidence.

#### Evidence Summary

A systematic review of the literature was conducted in order to summarize and appraise the available evidence on the effectiveness and safety of ultra-early surgical intervention.

*Evidence Related to Benefits*. Four studies explored the impact of ultra-early surgery on neurological outcomes using the following cutoffs: (i) ≤ 4 vs 4 to 24 hours; (ii) < 5 vs 5 to 24 hours; (iii) < 8 vs 8 to 24 hours; and (iv) < 12 vs 12 to 24 hours.^13,18,52,53^ Using an 8-hour threshold, patients treated ultra-early were 4.55 (95% CI 1.13 to 18.29) times more likely to improve by ≥ 2 grades on the AIS at 6 months than those treated early (8 to 24 hours).^53^ In contrast, a single study failed to detect a difference in the rate of improvement by ≥ 2 AIS grades at 6 months between patients treated within 4 hours of injury and those decompressed between 4 and 24 hours (RR .50, 95% CI .16 to 1.50).^52^ Given the limited evidence, it was not possible to make firm conclusions on the effectiveness of ultra-early surgery (<4 or <8 hours) in improving AIS by ≥ 2 grades at 6 months. The overall level of evidence for this outcome was very low. Two studies explored whether ultra-early surgery was associated with a *≥*2 grade improvement on AIS at 12 months.^13,18^ Using a 5-hour threshold, patients treated ultra-early were less likely to improve by *≥* 2 grades on AIS than those treated early (5 to 24 hours) (RR .24, 95% CI .07 to .85).^18^ Using a 12-hour threshold, there was no difference in the likelihood of achieving *≥*2 AIS grades at 12 months between the ultra-early (<12 hours) and early (12 to 24 hours) groups (RR 1.09, 95% CI .39 to 3.04).^13^ Given the results of these 2 studies, it was not possible to make definitive conclusions on the effectiveness of ultra-early surgery (<5 or <12 hours) in improving AIS by *≥* 2 grades at 12 months. The overall level of evidence for this outcome was also very low.

A single study compared the median improvement in AMS at 6 months between patients treated ultra-early (<8 hours) and those treated early (8 to 24 hours).^53^ Based on their results, the median improvement in AMS was 38.5 (95% CI 10.0 to 61.0) in the ultra-early group and 15.0 (95% CI 9.9 to 34.0) in the early surgery group.

Since the completion of the systematic review, an additional study was published investigating outcomes of decompression surgery performed ≤12 hours after SCI vs >12 hours.^54^ This study found a trend in favor of ultra-early decompression (≤12 hours) for LEMS change at 12 months; however, several methodological limitations render the results of this study challenging to interpret. Specifically, there were significant differences between the early and late surgical groups (particularly the baseline ASIA score), the study was underpowered, and there was a lack of reporting of clinically relevant outcome measures including UEMS for cervical SCI cases. It is therefore unlikely that results from this study would have changed the conclusions or recommendations.

*Evidence Related to Harms*. Two prospective cohort studies evaluated mortality rates in patients treated ultra-early (<4 hours or <8 hours) vs early (4 or 8 to 24 hours).^52,53^ In the study that evaluated a 4-hour cutoff, the mortality rate was 0 in both the ultra-early and early surgical groups.^52^ In the second study, mortality was also infrequent and did not differ between groups: 2/26 in the ultra-early group (<8 hours) and 1/22 in the early group (8 to 24 hours, RR 1.69, 95% CI .16 to 17.44).^53^ The overall level of evidence was low. A single study evaluated rates of neurological deterioration in patients undergoing surgery within 12 hours of injury compared to 12 to 24 hours.^13^ Only 3 patients experienced neurological deterioration in a cohort of 57: 2 in the ultra-early and 1 in the early group. There was no significant difference in the frequency of neurological deterioration based on the timing of surgical decompression (RR 1.56, 95% CI .15 to 16.27). The overall level of evidence for this outcome was very low.

#### Rationale for Recommendation

The outcomes ranked as critical for decision making were improvement of AIS by ≥ 2 grades and AMS, as well as any major complication, including mortality and neurological deterioration. As highlighted above, the overall quality of evidence for these critical outcomes was rated as very low. Similar to part 1, the GDG agreed that the main outcomes driving the recommendation for ultra-early surgery are improvement in AIS by ≥2 grades and improvement in AMS at >6 to 12 months. The GDG acknowledged that there is inconsistency with respect to the impact of ultra-early surgery on these critical outcomes depending on what time threshold was evaluated. Based on these discussions, the GDG voted that the overall certainty of the evidence was either very low (87%) or low (13%).

Similar to part 1, the GDG agreed that there was probably no (44%) or no (31%) important uncertainty or variability with respect to how much key stakeholders value the main outcomes.

The anticipated desirable effects of ultra-early surgical decompression include improvement of AIS by ≥ 2 grades and improvement of AMS. Based on very low evidence, it was not possible to make firm conclusions on the effectiveness of ultra-early surgery (<4, <5, <8, and <12) in improving AIS by ≥ 2 grades at 6- or 12 months.^13,18,52,53^ Given the limitations in the literature, 75% of the GDG stated that they did not know how substantial the desirable anticipated effects were for ultra-early surgery.

The anticipated undesirable effects of ultra-early surgery include mortality and neurological deterioration. Based on very low evidence, there were no differences in rates of mortality or neurological deterioration between patients treated ultra-early (<4, <8, or <12 hours) compared to early (4, 8, or 12 to 24 hours).^13,52,53^ Unfortunately, the majority of studies were underpowered to detect a difference in complications based on timing of surgery. Fifty-three percent of the GDG voted that the undesirable effects of very early surgery were likely trivial, 20% agreed they were small, and 27% stated they did not know. Given the desirable effects of ultra-early surgery were unknown and the undesirable effects were either trivial or small, the GDG agreed that the balance between the desirable and undesirable effects was unknown.

Although the management of SCI requires substantial resources, there were no studies that compared costs between patients treated ultra-early and early. The GDG, however, agreed that the resources required for ultra-early surgery were likely similar to the resources required for early surgery. Length of hospital stay was also considered when evaluating overall cost and resource requirement of ultra-early surgery. A single study by Jug et al (2015) determined that patients undergoing ultra-early (<8 hours) surgery had a shorter length of hospital stay (38.8 ± 24.0 days) than patients treated early (8 to 24 hours) (48.8 ± 40.3 days); however, this difference was not statistically significant (MD 10.0, 95% CI −30.31 to 10.31 days).^53^ Sixty-nine percent of the GDG agreed that the costs and savings associated with ultra-early surgery were negligible given that the resource requirements are likely similar for ultra-early and early surgery. An additional 23% of participants stated they did not know given the absence of strong literature. Ninety-two percent of the GDG voted that there were no studies that compared the resource requirements between ultra-early and early surgical decompression. Similarly, the cost-effectiveness of ultra-early compared to early surgery was largely unknown given the unknown neurological benefit associated with ultra-early surgery and the uncertainty surrounding the resource requirement. Sixty-percent of the GDG stated that they did not know the cost-effectiveness of ultra-early surgery while 40% voted that it does not favor either the intervention or the comparison.

Fifty-four percent of the GDG stated that they did not know the impact of a recommendation of ultra-early surgery on health inequities and 15% noted that they were uncertain. Furthermore, the GDG was uncertain whether a recommendation for ultra-early surgery would be acceptable to clinicians and patients with traumatic SCI due to unclear neurological benefit (uncertain: 62%, don’t know: 15%). Furthermore, it was uncertain whether payers would accept a recommendation of ultra-early surgery due to unclear costs and unknown potential for long-term savings. Finally, recommendations for some, if not all, of the time thresholds would be difficult to implement into clinical practice. In a study by Wilson et al (2016), the mean time from injury to arrival at a definitive care center was 8.1 ± 25.5 hours, while the mean time to surgery was 49.4 ± 65.0 hours.^55^ Moreover, only 34.2% reached the operating room within 12 hours. As such, pathways of care for SCI would need to be further streamlined in order to successfully implement a recommendation for ultra-early surgery. In addition, some patients require hemodynamic stabilization prior to undergoing surgery, creating further barriers to adopting a recommendation for ultra-early surgery. The voting with respect to the feasibility of ultra-early surgery was therefore split: 21% no, 21% probably no, 50% uncertain, and 7% varies.

Considering these factors, 100% of the GDG voted that the balance between the desirable and undesirable consequences was closely balanced or uncertain. As a result of this uncertainty, the GDG agreed that a recommendation for very early surgery could not be made at this time.

The final wording that a recommendation for ultra-early surgical decompression could not be made with the current body of evidence was subjected to a final vote of all the GDG members and consensus was achieved for its approval (>80%). Those who dissented either believed that the evidence did support the benefit of ultra-early decompression or had concerns about whether ultra-early decompression was feasible to achieve or practical.

#### Evidence Gaps and Future Recommendations

Through the development of these CPGs, we identified several important knowledge gaps and areas of future research:

1. *The impact of surgical timing across different SCI phenotypes, specifically acute CCS..* Little of the current evidence base on timing of surgery for SCI has specifically focused on the unique entity of CCS. Based on current evidence for other injury patterns and the few studies examining timing of surgery for CCS, it is reasonable to extrapolate that a similar benefit of early surgery is afforded in this patient population. However, as CCS is the most common pattern of incomplete SCI, with an increasing incidence particularly among the growing elderly population, further research on the impact of surgical timing on neurological recovery in these patients is required. It is recognized that since the completion of the systematic review and guideline process, at least 2 high-quality studies on the timing of surgery for CCS and incomplete cervical SCI have been published.^56,57^ Future efforts will need to address this new knowledge to update the current guidelines.
2. *The influence of SCI severity or level of injury on the treatment effect of early surgery.* While there are several studies that incorporate patients with various levels and severities of SCI, estimates have typically been pooled and thus conclusions cannot be made in specific patient subgroups. Future studies should be designed to determine the impact of timing of surgical decompression on neurological outcomes in different patient subgroups in order to better establish personalized treatment approaches and correctly triage patients.
3. *The influence of ultra-early surgery on patient-related outcome measures.* While there are a number of studies that have attempted to determine the impact of surgery performed earlier than 24 hours, the lack of consistent time frames in the literature has significantly hindered pooling of the evidence. Establishing consistent timing thresholds to be used in future studies is critical in order to definitively determine whether ultra-early surgery provides additional benefits to patients. Careful consideration must be made in future studies to the timing of the neurological examination during this ultra-early period. This indeed is an important limitation in the interpretation of neurologic benefit after ultra-early surgery. Patients undergoing ultra-early surgery must be neurologically assessed very early after their injury, when they still may be in spinal shock and when their examination is inherently less reliable at predicting outcome. And so, a person deemed “AIS A” when examined 2 hours post-injury who then has an immediate surgical decompression at 4 hours post-injury is not necessarily comparable to a person deemed “AIS A” when examined 14 hours post-injury who then has surgery at 20 hours post-injury. Accounting for this will be important in future studies that try to establish the true neurologic benefit of ultra-early surgery. Furthermore, studies investigating barriers to early surgical intervention and strategies to address them, particularly in under-resourced settings, are critical in ensuring adherence to established CPGs.
4. *Establishment of what constitutes a clinically meaningful improvement on the outcome measures used to evaluate neurological and functional status.* The development and incorporation of new metrics to assess functional improvement in future studies will aid in establishing clearer benchmarks for clinically meaningful improvements.
5. *The cost-effectiveness of early* vs *late surgery.* Since publication of the last set of guidelines in 2017, there has been only 1 additional study investigating the cost-effectiveness of early surgery. Nevertheless, there remains a paucity of evidence in this area which necessitates further research.
6. *The issue of what constitutes an effective decompression is an area of evolving interest.* The role of duroplasty, extent of spinal column decompression (eg, number of levels of laminectomy), surgical approach, and the use of intraoperative ultrasound to validate the effectiveness of decompressive surgery are areas of ongoing investigation that will require high-quality prospective comparative effectiveness studies to move the field forward.

#### Implementation Considerations

Dissemination of this guideline will be accomplished at multiple levels in order to ensure effective implementation into clinical practice:

1. Presentation at international spine surgery, critical care, neurology, and anesthesiology conferences.
2. Scientific and educational courses.
3. Online webinars that engages a broad audience in an interactive format.
4. Publication of a focus issue in the Global Spine Journal.
5. Submission to Emergency Care Research Institute (https://www.ecri.org/) for dissemination of these guidelines.

#### Internal Appraisal and External Review

The leader of the GDG and a methodologist from Aggregate Analytics completed an internal appraisal of the final guideline using the AGREE II checklist. A multidisciplinary group of stakeholders were invited to externally review this guideline document prior to publication. This guideline was also reviewed by several professional societies. The methods paper published elsewhere in this focus issue summarizes additional details of these processes and highlights the conflicts of interest of both internal and external reviews.

#### Plans for Updating

This guideline will be reviewed by AO Spine, Praxis Spinal Cord Institute, and members of the leadership group at 3 to 5 years following publication. The guideline will be updated at this time, or earlier, if there are changes in (i) the evidence related to benefits and harms, (ii) outcomes deemed critical for decision making, (iii) knowledge related to resource requirements and cost-effectiveness, and (iv) available interventions and resources.

## ORCID iDs

Michael G. Fehlings https://orcid.org/0000-0002-5722-6364

Laureen Hachem https://orcid.org/0000-0002-5886-9935

Nathan Evaniew https://orcid.org/0000-0003-1974-5224

Stephen L. McKenna https://orcid.org/0000-0003-2030-8988

Chris J. Neal https://orcid.org/0000-0002-5072-6454

Narihito Nagoshi https://orcid.org/0000-0001-8267-5789

Hiroaki Nakashima https://orcid.org/0000-0002-0039-9678

Ricardo Rodrigues Pinto https://orcid.org/0000-0002-6903-348X

Rex A. W. Marco https://orcid.org/0000-0003-3393-1672

Jefferson R. Wilson https://orcid.org/0000-0001-5965-0305

David E. Kahn https://orcid.org/0000-0002-9390-5938

Yi Lu https://orcid.org/0000-0001-8180-8578

Paul M. Arnold https://orcid.org/0000-0002-4622-7695
